# Stronger prediction of motor recovery and outcome post-stroke by cortico-spinal tract integrity than functional connectivity

**DOI:** 10.1371/journal.pone.0202504

**Published:** 2018-08-23

**Authors:** Leanne Y. Lin, Lenny Ramsey, Nicholas V. Metcalf, Jennifer Rengachary, Gordon L. Shulman, Joshua S. Shimony, Maurizio Corbetta

**Affiliations:** 1 Department of Radiology, University of Kentucky, Lexington, Kentucky, United States of America; 2 Physical Therapy Department, Carroll University, Waukesha, Wisconsin, United States of America; 3 Department of Neurology, Washington University School of Medicine, Saint Louis, Missouri, United States of America; 4 Mallinckrodt Inst. of Radiology, Washington University School of Medicine, Saint Louis, Missouri, United States of America; 5 Department of Bioengineering, Washington University School of Medicine, Saint Louis, Missouri, United States of America; Fraunhofer Research Institution of Marine Biotechnology, GERMANY

## Abstract

**Objectives:**

To examine longitudinal changes in structural and functional connectivity post-stroke in patients with motor impairment, and define their importance for recovery and outcome at 12 months.

**Methods:**

First-time stroke patients (N = 31) were studied at 1–2 weeks, 3 months, and 12 months post-injury with a validated motor battery and resting-state fMRI to measure inter-hemispheric functional connectivity (FC). Fractional anisotropy (FA) of the cortico-spinal tract (CST) was derived from diffusion tensor imaging as a measure of white matter organization. ANOVAs were used to test for changes in FC, FA, and motor performance scores over time, and regression analysis related motor outcome to clinical and neuroimaging variables.

**Results:**

FA of the ipsilesional CST improved significantly from 3 to 12 months and was strongly correlated with motor performance. FA improved even in the absence of direct damage to the CST. Inter-hemispheric FC also improved over time, but did not correlate with motor performance at 12 months. Clinical variables (early motor score, education level, and age) predicted 80.4% of the variation of motor outcome, and FA increased the predictability to 84.6%. FC did not contribute to the prediction of motor outcome.

**Conclusions:**

Stroke causes changes to the CST microstructure that can account for behavioral variability even in the absence of demonstrable lesion. Ipsilesional CST undergoes remodeling post-stroke, even past the three-month window when most of the motor recovery happens. FA of the CST, but not inter-hemispheric FC, can improve to the prediction of motor outcome based on early motor scores.

## Introduction

The World Health Organization has reported approximately 15 million cases of stroke worldwide, one third of which result in death, and another third which results in permanent disability [1]. The challenge for clinical neuroscience is to provide accurate prediction of functional impairment post-stroke to aid therapy and inform family. Traditionally, functional outcomes are determined by initial neurological deficit, age, and education level (as a proxy for socioeconomic status) [2, 3].

Another challenge is to define the mechanisms of neurological recovery at the systems level [4, 5]. For instance, it is clearly unknown if improvement of motor impairment is currently better explained by changes in structural (SC) or functional (FC) connections in the motor system. One animal (rat) study [6] that directly compared SC and FC measured with functional magnetic resonance imaging (fMRI) found that changes in fractional anisotropy (FA) using diffusion tensor imaging (DTI) to evaluate the integrity of axonal pathways were more sensitive than blood oxygen level dependent (BOLD) signals to assess functional connectivity (FC) with resting state functional magnetic resonance imaging (rsfMRI) [7–10].

The DTI metric fractional anisotropy (FA) is influenced by myelination, diameter, density, and orientation of axons. It can be used to measure white matter microstructure integrity and reorganization during stroke recovery, even in areas distant from injury [11, 12]. Post-stroke, FA values decline from Wallerian degeneration and recover over a period of weeks to months [4, 13, 14].

Multiple prior studies have demonstrated a correlation between FA and motor performance in stroke patients [8, 9, 15–19], including some longitudinal studies [13].

FC between inter-hemispheric homologous (homotopic) regions in the somatomotor network (SMN) have been also strongly correlated with deficits in motor performance [6, 20–22]. Longitudinally, patients with high inter-hemispheric functional connectivity should have better stroke recovery [21]. However, the combined influence of structural and functional connectivity on recovery is likely complex [23, 24].

Here we measure for the first time the longitudinal changes in CST-FA and inter-hemispheric FC in relation to motor recovery and examine imaging in relationship to motor performance during stroke recovery. Specifically, we are interested in defining whether metrics of SC or FC are more closely related to performance at different stages, and which metric is more sensitive to predict outcome, i.e. motor performance at 12 month.

## Material and methods

### Study population

Stroke subjects were prospectively recruited from the stroke service at Barnes-Jewish Hospital (BJH), with help from the Washington University Cognitive Rehabilitation Research Group (CRRG) (Dr. Lisa Connor) and the Stroke Trials Team (Dr. Jin Moo Lee) from 5/1/2008 to 5/30/2013. Inclusion criteria for stroke patients were: 1. Age ≥18. 2. First symptomatic stroke, ischemic or hemorrhagic etiology. 3. Up to two lacunes, clinically silent, less than 15 mm in size on CT scan. 4. Clinical evidence of motor, language, attention, visual, or memory deficits based on neurological examination. 5. Time of enrollment: <4 weeks from stroke onset. 6. Awake, alert, and capable of participating in research. Exclusion criteria were: 1. Inability to maintain wakefulness during testing. 2. Presence of other neurological, psychiatric or medical conditions that preclude active participation in research and/or may alter the interpretation of the behavioral/imaging studies (e.g. dementia, schizophrenia), or limit life expectancy to less than 1 year (e.g. cancer or congestive heart failure class IV). 3. Evidence of clinically significant periventricular white matter disease (grade ≥5) [25]. 4. Claustrophobia, which excludes patients from having an MRI study.

In total, 132 stroke patients were enrolled, and of these 57 had behavioral data on all 3 time points. This number was further reduced by selected only patients that had MRI scans at all 3 time points. This left 31 stroke patients (mean age 52.8 with range 22–77, 29 right handed, 14 female, 18 with stroke lesion overlapping the CST) which were enrolled for this study. Twenty healthy controls were also recruited and underwent identical exams. Inclusion criteria for control subjects were: Healthy adult matched to stroke study population by age, gender, handedness, and level of education. Exclusion criteria were: 1. Positive history of neurological, psychiatric, or medical abnormalities preventing participation in research activities. 2. History of atherosclerotic (coronary, cerebral, peripheral) artery disease. 3. Abnormal neurological examination.

All participants provided informed consent in accordance to the Washington University in Saint Louis Institutional Review Board which approved this study. All participants were compensated for their time. Clinical characteristics of participants were compared to source population for external validity.

### Behavioral testing and data reduction

Each subject underwent a neurobehavioral assessment including motor, language, attention, memory, and visual function at 1–4 weeks (1.9±0.7 weeks), 3 months (16.6±3.3 weeks), and 12 months post stroke (54.3±3.2 weeks) (S1 Table). Motor exams included measurements of active range of motion which included shoulder flexion, wrist extension/flexion, ankle flexion assessed in degrees using a goniometer [26], hand dynamometer [27], nine hole peg [28], action research arm test [29, 30], timed walk/functional independence measure [31], and the lower extremity motricity index [32], summarized with a principal component analysis (PCA) with oblique rotation (oblimin) (IBM Corporation, SPSS) to reduce the number of variables (S2 and S3 Tables) . Each component had to satisfy two criteria: (1) the eigenvalue had to be > 1; (2) the percentage of variance accounted for had to be > 10%. It should be noted that in order to provide a more comprehensive evaluation of our subjects we included behavioral testing results from both the upper and lower extremity.

### DTI acquisition and processing

DTI scans were obtained 3 and 12 months post stroke. All subjects were scanned with a Siemens 3.0T Tim-Trio scanner at Washington University School of Medicine. Structural scans were performed with T1-weighted MP-RAGE (1.0×1.0×1.0mm voxels; TE = 226ms, TR = 1950ms, flip angle = 9°), a transverse T2-weighted turbo spin echo sequence (1.0×1.0×1.0mm voxels; TE = 442ms, TR = 2500ms), and sagittal fluid attenuated inversion recovery (FLAIR) (1.5×1.5×1.5mm voxels; TR = 7500ms, TE = 326ms) [33]. DTI scans were collected in 48 directions using locally modified echo planar imaging (EPI) sequence (TR = 9600ms, TE = 92 ms, 2×2×2 mm voxels, *b* = 800–1200 s/mm^2^, total scan time 10.25 min). A template of the CST from the twenty healthy participants was created by overlapping the CST from all subjects in atlas space. The final template was selected from voxels that had a contribution from at least thirteen of the participants. Thirteen was picked qualitatively as a threshold that gave a reasonable approximation of the expected location and size of the CST. The lesion segmentation for the patients were used to create a conjunction map of the voxels within the CST but outside of the lesion [4, 33]. FA was averaged over these voxels for each patient and used for statistical analysis. This procedure was adopted because half of the subjects did not have lesions within the CST to analyze all subjects uniformly.

### rsfMRI acquisition and processing

At recruitment, three months, and 12 months post stroke, rsfMRI scans were obtained. Patients were instructed to fixate on a central cross projection. Scans were performed with a gradient EPI sequence (32 continuous 4mm slices; 4×4 in-plane resolution; TE = 27ms, TR = 2000ms), and ran for 4.4 min yielding 128 frames per run. Each patient received six to eight runs, and all runs were concatenated into a single data set. Preprocessing and atlas transformations were performed as previously described [34]. The regions of interest (ROIs) in the SMN along the M1 as previously defined were used for resting state analysis [35]. Pearson correlation coefficients (*r*) for homologous ROI pairs were calculated and Fisher *z* transform was applied to normalize values. The average of the *z* scores was the BOLD homotopic connectivity value used in statistical analysis.

### Statistics

Statistical analyses were carried out with Matlab version 7.12.0.635 (R2011a) and Statistical Analysis System (SAS) version 9.3. A simple linear multivariable regression was carried out using type III sums of squares with proc GLM (SAS version 9.3). The p-value for acceptance to the model was the default value of p<0.2. Traditional and imaging variables were used in a multivariable model to explain early behavioral deficits, and then those variables along with early behavioral deficits were correlated to chronic behavior. The first model used age, education level, and early motor factor scores to predict 12 month contralateral motor factor score. The second model used FA ratio (ratio of ipsilesional to contralesional FA) and BOLD homotopic connectivity to predict 12 month contralateral motor factor score. The last model included all of the above measures.

## Results

### Patients and lesion anatomy

31 patients with acute stroke representative of the stroke source population were studied (**Table 1**). Distributions of lesion were assessed by a board certified neurologist (MC) **(S1 Fig)**; 18 lesions overlapped with the CST (average overlap with CST is 13.24%). The CST was defined as ipsilesional or contralesional depending on the side of the stroke.

**Table 1:** Patient characteristics.

ID	Lesion side	Age	NIH-SS	White matter disease	Lacunar infarcts	Lesion site	Lesion type	Lesion size (cm^3^)	%CST overlap
051	Right	56	4	1	2	subcortical	Ischemic	0.83	20.2
056	Right	52	3	0	3	cortical	Ischemic	11.1	1.6
058	Right	53	4	1	0	subcortical	Ischemic	2.35	22.1
060	Left	56	7	2	0	brainstem	Ischemic	2.97	19.9
065	Right	50	6	0	0	brainstem	Ischemic	0.95	0
067	Left	54	4	5	2	white matter only	Ischemic	0.94	28.0
071	Right	64	5	0	2	brainstem	Ischemic	0.10	0
083	Left	60	1	2	1	cortical	Ischemic	2.68	0
084	Right	51	1	0	2	cortical	Ischemic	2.58	0
088	Left	54	12	5	15	subcortical	Hemorrhagic	23.0	3.8
090	Right	52	5	0	1	brainstem	Ischemic	0.73	28.6
092	Left	66	4	0	0	cortical	Ischemic	3.76	1.3
097	Left	44	6	0	0	cortical	Ischemic	6.17	0.4
099	Left	54	13	0	0	cortico-subcortical	Ischemic	111.0	2.0
101	Right cerebellar	58	3	0	2	cerebellar	Ischemic	16.1	0
102	Left	38	4	0	0	subcortical	Ischemic	1.29	0
104	Left	68	4	6	2	white matter only	Ischemic	1.30	32.1
105	Left	22	1	0	0	cortico-subcortical	Hemorrhagic	32.1	0
108	Left	70	4	1	1	cortical	Ischemic	50.3	0
109	Right	40	0	1	1	cortico-subcortical	Ischemic	118.0	0.3
111	Right	56	12	1	2	white matter only	Ischemic	13.7	8.7
112	Left	64	11	5	1	subcortical	Ischemic	9.80	21.8
119	Left	61	-	1	1	cortical	Ischemic	31.2	0
120	Left	77	-	1	1	cortical	Ischemic	22.6	0
122	Left	64	8	1	1	cortical	Ischemic	55.8	0
124	Left	41	-	1	2	cortical	Ischemic	2.59	0
133	Right	62	10	1	0	cortical	Ischemic	75.8	0
138	Right	40	1	0	2	subcortical	Ischemic	22.2	5.9
140	Right	40	12	0	0	cortico-subcortical	Hemorrhagic conversion	82.4	4
142	Left	30	8	2	0	subcortical	Ischemic	3.20	14.8
145	Right	40	-	0	0	subcortical	Ischemic	1.53	22.9

### Motor performance and recovery

The PCA yielded two factors accounting for 75% of the variability of motor scores across subjects. Tests for the left body loaded on one factor, while tests for the right body loaded on another factor. Therefore the two motor factors provided a continuous measure of motor performance highly correlated with the NIHSS (**S2 Fig**), which allow for greater sensitivity in correlation analyses.

**Fig 1:**
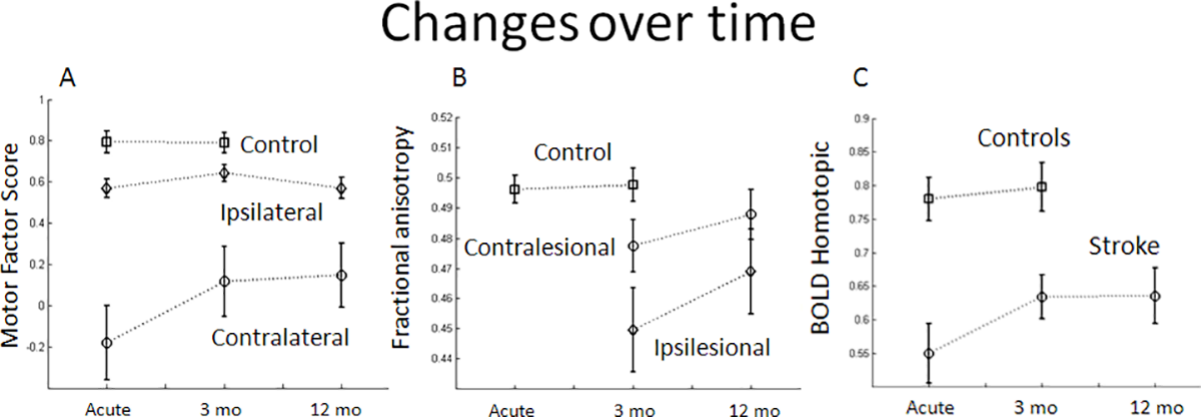
Time course of changes in the motor factor scores, fractional anisotropy of the CST, and BOLD homotopic FC of the SMN.

Motor factor scores in healthy controls were stable across two testing sessions conducted three months apart, showing the reproducibility of the factor scores. In stroke patients, at the sub-acute stage, contralateral motor scores were lower than ipsilateral scores (*p*<10^−4^), and both were lower than motor scores in healthy subjects (**Fig 1A**). Over time, there was significant improvement of motor performance on the contralateral side with significant interaction of Time by Side (*p* = 8x10^-4^). At three months, contralateral motor scores had improved (*p* = 4.4x10^-3^), but remained significantly lower than controls. At twelve months, contralateral motor scores remained lower than ipsilateral scores (*p* = 5x10^-3^). Interestingly, although ipsilateral motor performance across the three time points was lower than healthy controls (*p*<10^−4^), it did not change during the year post-stroke (*p* = 0.9858) (**Fig 1A**).

Both contralateral and ipsilateral motor scores were strongly correlated across time points. A similar percentage of patients improved from the early time point to 3 months in motor performance (70.97% contralateral; 64.52% ipsilateral).

### Diffusion imaging and motor performance

The FA values in the CST of healthy controls were stable across sessions separated by three months (**Fig 1B**). In stroke subjects, the 3-month ipsilesional CST-FA was significantly lower than those in controls and contralesional FA (*p* = 0.0168); Interestingly, ipsilesional FA was highly correlated with the contralateral motor factor score even for the 13 subjects who did not have lesions overlapping the CST (*p* = 1.4x10^-4^ at 3 months). The contralesional FA was not significantly different from controls.

**Fig 2:**
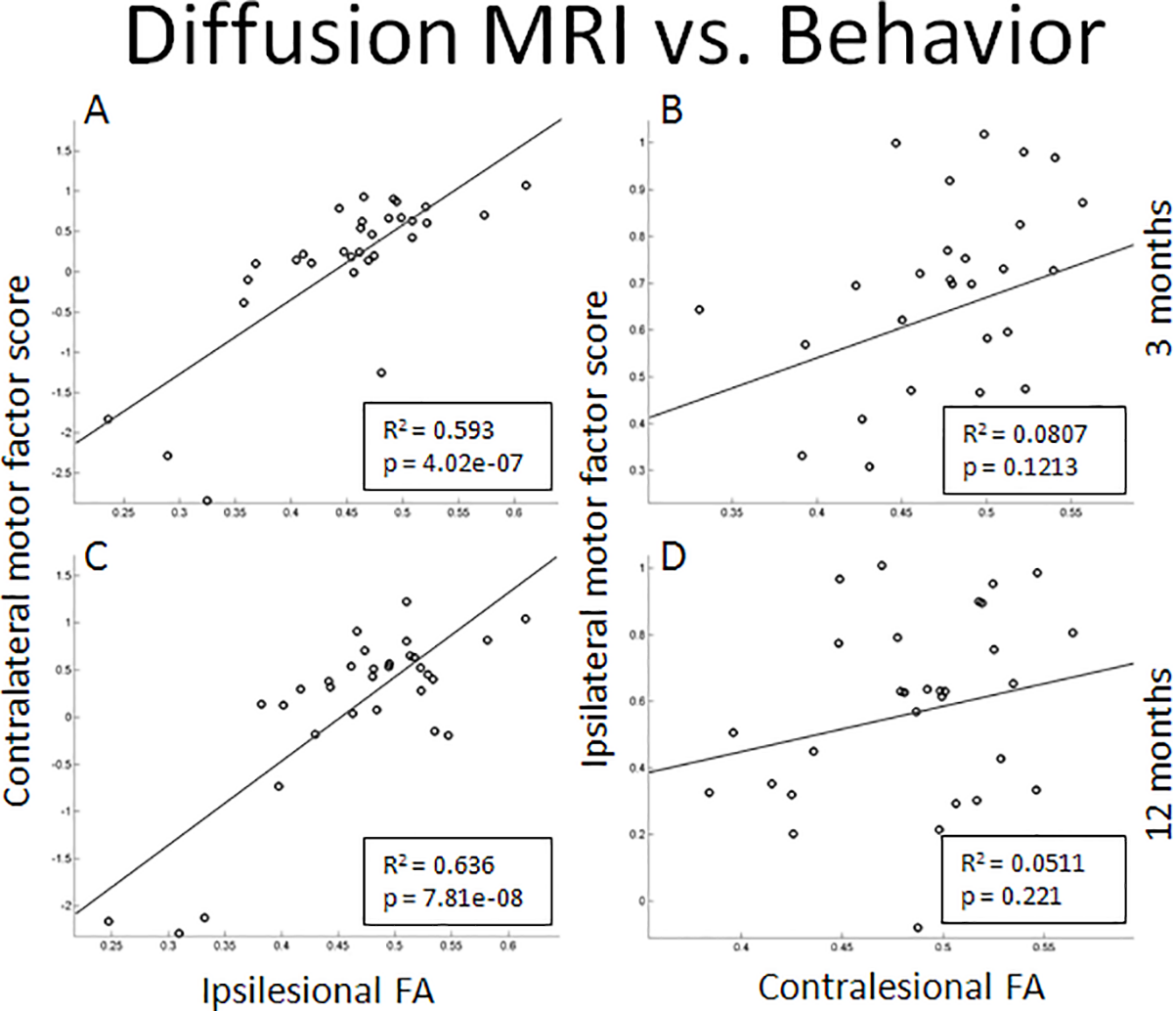
Correlation between the FA of the CST (outside of the lesion) and the motor factor score of the corresponding side at 3 months and 12 months. The correlation of FA to factor score is significant on the abnormal side, and has a wider range of values than the normal side.

CST-FA values significantly improved in stroke subjects from 3 to 12 months, especially ipsilesionally, as captured by a significant interaction of time by side (*p* = 3.9x10^-3^) (**Fig 1B**). There was also a significant change in FA ratio between three and twelve months (*p* = 2.8x10^-3^); this latter result accounts for the significant change in the ipsilesional FA (*p* = 4.2x10^-3^) compared to the non-significant change in contralesional FA (*p* = 0.0887). At 12 months, ipsilesional FA was no longer significantly different from controls, and there was no difference between ipsilesional and contralesional FA (*p* = 0.0958) (**Fig 2B and 2C**).

Ipsilesional FA values were significantly correlated with the contralateral motor factor score at both time points (**Fig 2A and 2C).** Contralesional FA (normal side) was not significantly correlated with the corresponding ipsilateral (normal side) motor factor score (**Fig 2B and 2D)**, but was correlated with the contralateral motor factor (abnormal side) score (*p* = 0.034885).

### Functional connectivity

**Fig 3:**
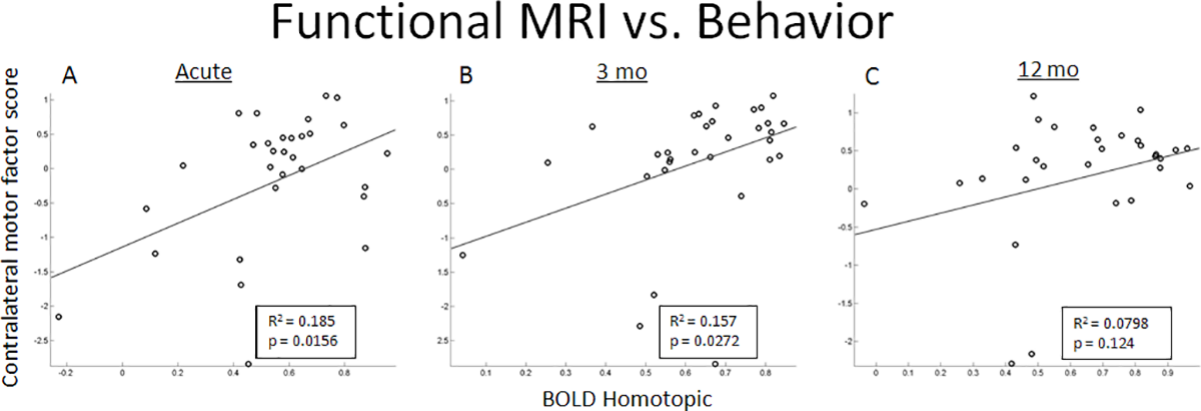
Correlation between the BOLD homotopic connectivity of the SMN and the motor factor score of the corresponding side at recruitment (early), 3 months, and 12 months. The correlation of FA to factor score is significant at the early time period and the 3 month time point.

In healthy subjects homotopic motor FC was stable across two sessions three months apart (*p* = 0.5332). In stroke patients at the early stage, homotopic FC was significantly decreased compared to controls, and increased in the first three months (*p* = 0.0259), but did not change further at 12 months (**Fig 1C**). Homotopic FC was strongly correlated with the contralateral motor factor score at the early and 3 month time periods, but not at the 12 month time period **(Fig 3A and 3C)**.

### Multivariable regression

FA was significantly correlated with amount of CST damage (defined by number of voxels in the CST that is within the lesion) (*p* = 2.1x10-4). A significant portion of the subjects (13 total) did not have lesions overlapping the CST, so both the within lesion FA and lesion size were left out of the final regression model, although their ipsilesional FA is still highly correlated with the contralateral motor factor score (*p* = 1.4x10-4 at 3 months).

The predictive multivariable regression showed traditional predictors (age, education level, and early deficits) accounted for more variation of the 12-month contralateral motor factor score (*R*^*2*^ = 0.804), than the imaging metrics (BOLD homotopic FC and FA ratio) (*R*^*2*^ = 0.654). When accounting for the FA ratio, the BOLD homotopic FC was not significantly associated with the 12-month motor factor score (*p* = 0.9591), yet while accounting for BOLD homotopic FC, the FA ratio is still significantly associated with the 12-month motor factor score (*p* = <0.0001). With the traditional and imaging metrics combined, only the early motor factor scores (early deficits) and FA ratio remained in the model as significant predictors of the 12-month motor factor score (**Table 2**). The final model predicted 12-month deficits better than the traditional predictors (*R*^*2*^ = 0.8469).

**Table 2:** Multivariable linear regression predicting 12 month factor score.

Predictors	Clinical model	Imaging model	Combined model
Age	0.053	—	0.1350
Education level	0.0707	—	0.2696
Early motor factor score	< .0001	—	< .0001
Early BOLD Homotopic	—	0.9591	0.9344
3 mo FA ratio	—	< .0001	0.0145
Total	< .0001	< .0001	< .0001
R^2^	0.804	0. 654444	0.846404

## Discussion

As far as we know, this is the first human study looking at both DTI and FC changes in stroke patients longitudinally. Another unique aspect of our study is that in order to provide a more comprehensive evaluation of our subjects we included neurobehavioral results from both the upper and lower extremity in a combined factor score. Both DTI and FC show changes over time highly correlated with motor recovery, even past the first three months, with DTI being more predictive of chronic motor performance. These findings parallel animal studies looking at DTI and FC changes longitudinally that have also reported final sensorimotor performance outcome is better predicted by measures of DTI than inter-hemispheric FC [36].

### Behavior

Most motor recovery occurred in the first 3 months post-stroke, consistent with previous work [37]. Correlation among motor scores is also consistent with previous work showing deficits in one domain (e.g. range of motion) are correlated with deficits in another domain (e.g. hand function) [38, 39]. In addition, ipsilateral (to the stroke) motor performance was below control levels, and did not improve over time, which is consistent with abnormalities of ipsilateral arm kinematics described in stroke [40].

### Structural vs. functional connectivity and motor recovery

Previous studies documented a strong relationship between CST-FA and motor deficits in chronic stroke patients. One study reported both ipsilesional and contralesional CST play a role in motor recovery after unilateral stroke [8]. Van Meer et al. documented longitudinal FA improvements in stroke patients [36].

We showed the amount of damage in CST from 3 to 12 months post-stroke is strongly correlated with motor performance. Even in patients with no direct structural damage to the CST, there are FA changes in the CST that correlate with motor deficits. These findings suggest stroke causes changes to the CST microstructure that can account for behavioral variability even in the absence of demonstrable lesion. They also indicate ipsilesional CST undergoes remodeling post-stroke, even past the three-month window when most of the motor recovery happens. One explanation for this observation is indirect microstructural damage due to disuse.

DTI scans were not obtained at the early time point because of concern that the reliability of the measure could be affected by rapidly changing diffusion values due to edema, and previous evidence indicating the FA values of the affected CST decline over weeks post-stroke [13]. However, our findings clearly indicate FA improvements occur past the 3-month time point, and since motor recovery was still not complete, the 3-month DTI scan was a good proxy for the early CST tract damage. In fact, at both time points, the FA values on the damaged side were correlated with contralateral motor performance. The CST on the undamaged hemisphere showed insignificant change over time, and decrease in FA values was within normal variability.

Homotopic FC between motor regions also improved longitudinally, but mostly within the first three months post stroke, which significantly correlated with motor performance during the same time periods, but not at 12 months after stroke. This indicates the recovery of FC happens mostly within three months post stroke, and never reaches the same level as the controls.

### Prediction of motor outcome

FA ratio (ipsilesional/contralesional CST) can provide valuable prognostic information of chronic motor performance in addition to initial motor impairment. In the multivariable linear regression, imaging metrics (homotopic motor FC, FA ratio) alone accounted for less variability in 12-month motor factor score than traditional metrics (age, education, and early deficits). However, in the combined model, age and education level were no longer significant after taking into account the effect of FA ratio. This suggests the microstructural integrity of the CST accounts for some of the baseline patient characteristics which age and education level approximates.

The strong predictive value of initial impairment is well known and may be related to structural and functional factors. Both damage to the CST and inter-hemispheric FC have been correlated with initial impairment. Ultimately, FC was not significant in the multivariable regression for 12-month functional outcomes. This result may indicate that inter-hemispheric FC is influenced by the integrity of CST axonal tracts.

A notable observation from our results is that a simple model based only on clinical information (age, education, early motor score) provides nearly identical correlation value as compared to the full model with imaging information as gauged by the R^2^ value.

### Limitations

Limitations of the current study include a small sample size, although it should be noted that we required that all subjects had the full complement of neurobehavioral testing and MRI scan at all 3 time points. This requirement also shifted the average age of our group towards younger patients with milder strokes. An additional limitation of the study was the absence of DTI at the acute time point. We purposely chose to shorten the length of the first MRI scan anticipating that the patients will be sicker at this early time point and uncomfortable during the scan.

## Conclusion

Stroke causes changes to the CST microstructure that can be measured by the FA values and account for variability in motor scores even if the lesion itself does not involve the CST. The remodeling of the CST is demonstrated by the improved FA from 3 to 12 months post stroke, beyond the traditional three month recovery period post stroke. Homotopic FC between motor regions also show improvement over time, and is correlated with motor performance; however, it is very colinear with FA values and therefore does not offer any additional information in explaining the variability of motor performance. From the regression analysis, FA can objectively quantify baseline patient characteristics previously measured using proxy variables such as age and education level and be used in addition to early motor scores to predict motor outcomes. Based on our current results it would be interesting to investigate FA changes during recovery in other projection fiber tracks and related networks beyond the motor system such as vision, language, or even executive control areas.

## Supporting information

S1 Fig Color coded distribution of lesion location in the brain. The cortical spinal tract is marked in light blue. Table 1 gives more information on lesion location.
(TIF)

S2 Fig Correlation of motor factor scores to NIHSS early (circles) and 3 months (X’s). The correlation is significant at both time points (p = 1.9 × 10^−11^ and 1.3 × 10^−10^, respectively).
(TIF)

S1 Table Time from stroke when all tests were performed (in days). (DOCX)

S2 Table Motor factor scores at the 3 time points. (DOCX)

S3 Table Weights assignments for factor scores. (DOCX)
