# Supplementary figures and images for: Stronger prediction of motor recovery and outcome post-stroke by cortico-spinal tract integrity than functional connectivity
Source: PLoS One. 2018 Aug 23;13(8):e0202504. doi: 10.1371/journal.pone.0202504 (PMC6107181; doi:10.1371/journal.pone.0202504)

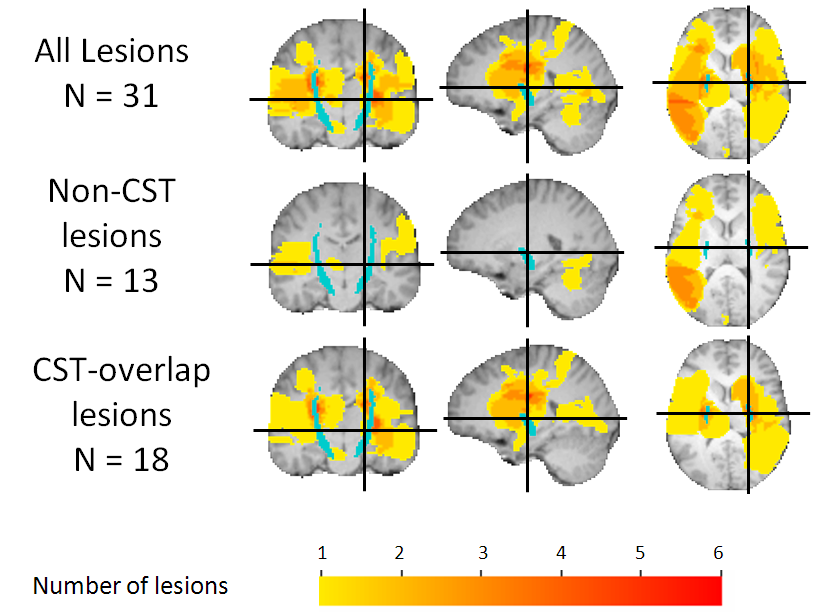

Supplement: S1 Fig — The cortical spinal tract is marked in light blue. Table 1 gives more information on lesion location. (TIF) [file pone.0202504.s001.tif]

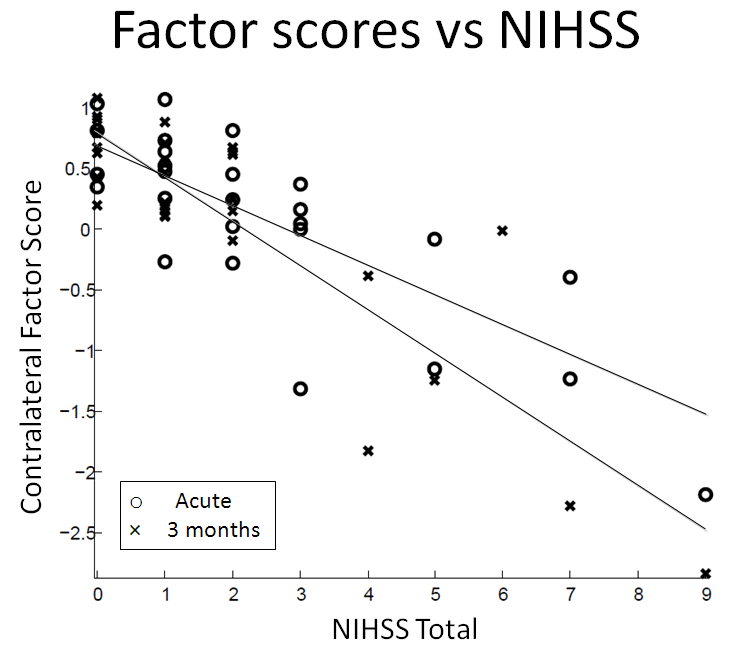

Supplement: S2 Fig — The correlation is significant at both time points (p = 1.9 × 10^−11^ and 1.3 × 10^−10^, respectively). (TIF) [file pone.0202504.s002.tif]
