# Supplementary material for: Stronger prediction of motor recovery and outcome post-stroke by cortico-spinal tract integrity than functional connectivity
Source: PLoS One. 2018 Aug 23;13(8):e0202504. doi: 10.1371/journal.pone.0202504 (PMC6107181; doi:10.1371/journal.pone.0202504)
Supplement: S1 Table — (DOCX) [file pone.0202504.s003.docx]

**Supplementary Table S1:** time from stroke when all tests were performed (in days)

| ID | Early Behavioral Test(days) | Early  MRI (days) | 3 month  Behavioral  Test(days) | 3month MRI (days) | 1 year Behavioral Test(days) | 1 year  MRI (days) |
| --- | --- | --- | --- | --- | --- | --- |
| 051 | 12 | 12 | 104 | 104 | 376 | 376 |
| 056 | 10 | 10 | 105 | 105 | 396 | 396 |
| 058 | 10 | 10 | 112 | 112 | 371 | 371 |
| 060 | 10 | 10 | 100 | 100 | 379 | 396 |
| 065 | 8 | 8 | 100 | 100 | 410 | 410 |
| 067 | 11 | 11 | 102 | 102 | 349 | 349 |
| 071 | 12 | 12 | 103 | 103 | 355 | 355 |
| 083 | 11 | 11 | 106 | 106 | 390 | 390 |
| 084 | 14 | 14 | 134 | 134 | 399 | 399 |
| 088 | 12 | 12 | 167 | 167 | 379 | 379 |
| 090 | 12 | 12 | 117 | 117 | 349 | 349 |
| 092 | 10 | 10 | 85 | 85 | 374 | 374 |
| 097 | 6 | 6 | 98 | 98 | 408 | 408 |
| 099 | 6 | 6 | 98 | 98 | 349 | 349 |
| 101 | 9 | 9 | 100 | 100 | 358 | 358 |
| 102 | 13 | 13 | 101 | 101 | 360 | 360 |
| 104 | 12 | 12 | 150 | 150 | 384 | 384 |
| 105 | 18 | 18 | 130 | 130 | 381 | 381 |
| 108 | 22 | 17 | 107 | 107 | 465 | 456 |
| 109 | 20 | 20 | 138 | 138 | 375 | 375 |
| 111 | 16 | 16 | 107 | 107 | 386 | 386 |
| 112 | 15 | 15 | 120 | 120 | 380 | 380 |
| 119 | 12 | 12 | 103 | 103 | 354 | 354 |
| 120 | 17 | 17 | 115 | 115 | 374 | 374 |
| 122 | 14 | 14 | 105 | 105 | 372 | 372 |
| 124 | 7 | 7 | 181 | 181 | 404 | 404 |
| 133 | 12 | 12 | 97 | 97 | 385 | 385 |
| 138 | 11 | 11 | 106 | 106 | 380 | 380 |
| 140 | 22 | 19 | 167 | 167 | 376 | 376 |
| 142 | 15 | 15 | 124 | 124 | 374 | 374 |
| 145 | 27 | 27 | 125 | 125 | 384 | 384 |
| Avg | 13.1 | 12.8 | 116.4 | 116.4 | 379.8 | 380 |
| Std | 4.8 | 4.4 | 23.1 | 23.1 | 22.8 | 22.0 |
