# Supplementary material for: Stronger prediction of motor recovery and outcome post-stroke by cortico-spinal tract integrity than functional connectivity
Source: PLoS One. 2018 Aug 23;13(8):e0202504. doi: 10.1371/journal.pone.0202504 (PMC6107181; doi:10.1371/journal.pone.0202504)
Supplement: S2 Table — (DOCX) [file pone.0202504.s004.docx]

**Supplementary Table S2:** Motor factor scores at 3 time points

| ID | Early Motor  Left Factor | Early Motor  Right Factor | 3 mo. Motor  Left Factor | 3 mo. Motor  Right Factor | 1 yr. Motor  Left Factor | 1 yr. Motor  Right Factor |
| --- | --- | --- | --- | --- | --- | --- |
| 051 | 0.33 | 0.63 | 0.18 | 0.39 | 0.45 | 0.73 |
| 056 | -0.46 | 0.87 | -1.16 | 0.77 | -0.32 | 0.78 |
| 058 | 0.12 | 0.55 | 0.30 | 0.79 | - | - |
| 060 | 0.39 | -0.39 | 0.46 | 0.32 | 0.35 | 0.43 |
| 065 | -1.27 | 0.39 | -0.27 | 0.75 | -0.39 | 0.53 |
| 067 | 0.77 | 0.15 | 0.77 | 0.46 | 0.77 | 0.58 |
| 071 | -0.29 | 0.35 | 0.21 | 0.51 | 0.11 | 0.44 |
| 083 | 0.70 | 0.70 | 0.72 | 0.87 | 0.42 | 0.52 |
| 084 | 0.81 | 0.81 | 0.74 | 0.77 | 0.63 | 0.62 |
| 088 | -0.22 | -1.36 | 0.37 | 0.33 | 0.42 | 0.24 |
| 090 | 0.34 | 0.68 | 0.49 | 0.75 | 0.52 | 0.56 |
| 092 | 0.78 | 0.26 | 0.87 | 0.79 | 0.89 | 0.78 |
| 097 | 0.83 | 0.42 | 1.00 | 0.90 | 0.92 | 0.98 |
| 099 | 0.22 | -2.87 | 0.43 | -2.20 | 0.48 | -2.36 |
| 101 | 0.63 | 0.56 | 0.59 | 0.60 | 0.66 | 0.55 |
| 102 | 0.34 | -1.20 | 0.60 | 0.15 | 0.74 | 0.40 |
| 104 | 0.16 | -0.35 | 0.45 | 0.22 | 0.28 | -0.04 |
| 105 | 0.87 | 0.93 | 0.83 | 0.92 | 0.90 | 0.93 |
| 108 | 0.33 | 0.16 | 0.72 | 0.71 | 0.43 | 0.38 |
| 109 | 0.79 | 0.88 | 0.79 | 0.84 | 0.80 | 0.77 |
| 111 | -2.33 | 0.41 | -2.00 | 0.62 | -2.33 | 0.26 |
| 112 | 0.55 | -2.09 | 0.42 | -2.87 | 0.29 | -1.88 |
| 119 | 0.54 | 0.50 | 0.57 | 0.64 | 0.55 | 0.49 |
| 120 | 0.25 | 0.36 | 0.48 | 0.36 | 0.48 | 0.27 |
| 122 | 0.59 | 0.53 | 0.42 | 0.30 | - | - |
| 124 | 0.68 | 0.74 | 0.77 | 0.68 | 0.74 | 0.73 |
| 133 | 0.11 | 0.62 | 0.22 | 0.49 | - | - |
| 138 | 0.39 | 0.41 | 0.65 | 0.73 | 0.55 | 0.61 |
| 140 | -1.68 | 0.59 | -0.34 | 0.77 | -0.62 | 0.73 |
| 142 | 0.62 | -0.34 | 0.82 | 0.77 | 0.83 | 0.77 |
| 145 | 0.80 | 0.72 | 0.74 | 0.68 | 0.65 | 0.73 |
