# Supplementary material for: Stronger prediction of motor recovery and outcome post-stroke by cortico-spinal tract integrity than functional connectivity
Source: PLoS One. 2018 Aug 23;13(8):e0202504. doi: 10.1371/journal.pone.0202504 (PMC6107181; doi:10.1371/journal.pone.0202504)
Supplement: S3 Table — (DOCX) [file pone.0202504.s005.docx]

**Supplementary Table S3:** Weights assignments for Factor Scores

| Behavioral test | Component 1 | Component 2 |
| --- | --- | --- |
| Shoulder flexion L | .954 |  |
| Shoulder flexion R |  | .903 |
| Wrist extension L | .893 |  |
| Wrist extension R |  | .857 |
| Dynamometer hand L | .779 |  |
| Dynamometer hand R |  | .731 |
| Nine hole peg L | .871 |  |
| Nine hole peg R |  | .837 |
| ARA Total L | .954 |  |
| ARA Total R |  | .916 |
| Timed Walk/FIM | .629 | .600 |
| Motoricity L | .901 |  |
| Motoricity R |  | .882 |
| Ankle range of motion L | .829 |  |
| Ankle range of motion R |  | .644 |
| Shoulder flexion L | .954 |  |
